# Biorefining of Walnut Shells into Polyphenol-Rich Extracts Using Ultrasound-Assisted, Enzyme-Assisted, and Pressurized Liquid Extraction Coupled with Chemometrics

**DOI:** 10.3390/foods14132245

**Published:** 2025-06-25

**Authors:** Busra Acoglu Celik, Muhammed Alpgiray Celik, Laura Jūrienė, Jovita Jovaišaitė, Rita Kazernavičiūtė, Erturk Bekar, Perihan Yolci Omeroglu, Petras Rimantas Venskutonis, Senem Kamiloglu

**Affiliations:** 1 Department of Food Engineering, Faculty of Agriculture, Bursa Uludag University, Gorukle Campus, 16059 Bursa, Türkiye; busraacoglu@gmail.com (B.A.C.); erturk@uludag.edu.tr (E.B.); pyomeroglu@uludag.edu.tr (P.Y.O.); 2 Department of Food Hygiene and Technology, Faculty of Veterinary Medicine, Bursa Uludag University, Gorukle Campus, 16059 Bursa, Türkiye; alpgiraycelik@gmail.com; 3 Department of Food Science and Technology, Kaunas University of Technology, Radvilėnų pl. 19, LT-50254 Kaunas, Lithuania; laura.juriene@ktu.lt (L.J.); jovita.jovaisaite@ktu.lt (J.J.); rita.kazernaviciute@ktu.lt (R.K.); rimas.venskutonis@ktu.lt (P.R.V.)

**Keywords:** *Juglans regia* L. shell, green extraction, combined extraction, phenolic compounds, antioxidant capacity, principal component analysis

## Abstract

Walnut (*Juglans regia* L.) shells are valuable agro-industrial by-products rich in polyphenols. This study investigated traditional (maceration) and advanced extraction techniques—ultrasound-assisted extraction (UAE), enzyme-assisted extraction (EAE), pressurized liquid extraction (PLE), and combined ultrasound–enzyme extraction (US-EAE)—to recover bioactive compounds from walnut shells. Extraction efficiency, total phenolic content (TPC), antioxidant capacity (ABTS^•+^, DPPH•), and polyphenol composition were evaluated. UPLC-ESI-MS/MS identified key polyphenols including ellagic acid, 4-hydroxybenzoic acid, vanillin, taxifolin, and quercitrin. The highest TPC (5625 mg GAE/100 g dw) was found in extracts subjected to US-EAE, in which ultrasound pretreatment (200 W, 10 min) was followed by enzymatic extraction using 0.06 mL/g Viscozyme^®^ L at pH 3.5 and 45 °C. Under the same extraction conditions, UAE alone yielded the second highest TPC (4129 mg GAE/100 g dw). The highest ABTS^•+^ scavenging activity (14,478 mg TE/100 g dw) and enhanced DPPH• activity (45.38 mg TE/100 g dw) were also observed in US-EAE extracts. Chemometric techniques (PCA and HCA) revealed meaningful clustering and variation patterns among methods. These findings highlight the potential of walnut shells as a sustainable source of polyphenols and demonstrate the effectiveness of innovative extraction technologies in maximizing bioactive compound recovery for potential functional applications.

## 1. Introduction

Agro-food by-products, including peels, stems, leaves, husks, shells, and kernels, are residual plant materials produced during industrial processing. These materials present a significant challenge for processed food manufacturers in terms of waste disposal, as they can have substantial adverse effects on the ecosystem. Due to the abundant bioactive composition found in agro-industrial wastes, there has been a notable emphasis on harnessing their potential for secondary applications [1]. These wastes present an exceptional opportunity as a source of value-added products, enabling cost-effective utilization across various nutraceutical domains [2].

In recent years, interest in healthy eating has increased with the identification of the relationship between diet and health. Various dietary recommendations have been developed to delay aging and reduce the risk of certain health problems such as cardiovascular diseases. Walnuts (*Juglans regia* L.) contain high concentrations of polyunsaturated fatty acid-rich oil, proteins, dietary fibers, sterols, folates, tannins, and polyphenolic antioxidants with health-promoting properties [3].

*Juglans regia* L. is acknowledged as a rich source of valuable chemicals. Within this context, the phytochemical constituents present in walnuts and their products (shell, green shell, leaf, root) display a diverse range of biological activities, including laxative, anti-proliferative, antibacterial, antiseptic, antifungal, anticancer, diuretic, antioxidant, and anti-inflammatory effects. Walnut products that positively affect human health are considered an excellent food source [3].

China holds the title of the world’s foremost walnut producer, accounting for approximately 50% of global production, trailed by the United States with approximately 25%. Other significant producers include Iran, Türkiye, Mexico, Ukraine, Chile, Uzbekistan, and France [4]. Worldwide production of walnut has exhibited consistent growth, reaching 2.67 million metric tons during the 2022/23 season [5].

Walnuts are cultivated worldwide for their edible kernels contained in a hard shell. The shell accounts for more than 50% of the total weight of the walnut, and about 2.3 million tons of walnut shell, which is agro-industrial waste, are generated annually, creating environmental waste and often being burned as fuel. The shell’s composition of lignin (52.3%), cellulose (25.5%), and hemicellulose (22.2%) makes it tough, non-toxic, biodegradable, and renewable [6].

The United Nations has published a universal call to action that includes 17 goals that member states aim to achieve by the end of 2030. One of these goals includes supporting the reduction in waste by industry, business, and consumers [7]. For this reason, it is of great importance to examine waste by-products such as walnut shells.

To minimize environmental impact and enhance the value of this by-product, the efficient utilization of walnut shells and alternative applications to obtain functional components have recently been of great importance [6]. Currently, there is a growing emphasis on the exploration of low-value by-products and waste from the food, forestry, and agricultural sectors, driven by both economic and environmental advantages. This focus aims to extract bioactive components from these sources for various purposes.

There have been different types of conventional and innovative extraction techniques available for the recovery of bioactive compounds from food waste and by-products [8,9]. Whereas traditional techniques include Soxhlet extraction, hydrodistillation, and maceration, innovative techniques—pressurized liquid extraction (PLE), ultrasound-assisted extraction (UAE), enzyme-assisted extraction (EAE), microwave-assisted extraction (MAE), pulsed electric field (PEF), high hydrostatic pressure (HHP), supercritical fluid extraction (SFE), and their combinations—belong to the advanced extraction techniques. Conventional extraction methods may exhibit certain drawbacks, including low process efficiency, high energy and time consumption, utilization of toxic solvents, thermal degradation of compounds, adverse environmental effects, and production of low-quality extracts [10]. Hence, innovative technologies provide shorter extraction times, increased extraction yields, and the use of eco-friendly solvents, ensuring the complete removal of the extracted substance [11].

Furthermore, these techniques are scalable and offer advantages over conventional extraction methods when combined with other processes in biorefinery approaches [12,13]. The biorefinery concept is of particular interest today due to the increasing efforts to reduce or eliminate industrial waste and the need for greener extraction technologies. This method is based on the application of a sequential extraction process that eliminates the need to manipulate biomass while efficiently recovering a wide range of compounds. Additionally, this approach highlights the potential to achieve a comprehensive biorefinery of by-products through the regulation of extraction parameters and the combined use of different technologies. According to these developments, the innovative extraction techniques mentioned above meet the criteria [13].

Recently, several papers were published detailing the application of innovative extraction techniques to walnut shells. Herrera et al. [14] applied the innovative technique of accelerated extraction in hazelnut and walnut shells. In the study, lipophilic and hydrophilic compounds were extracted and the main chemical constituents in the hydrophilic fraction of walnut shells were identified as sugar derivatives, phenolic compounds, resin acids, fatty acids, and flavonoids, from highest to lowest. Han et al. [15] determined the best method by analyzing total phenols in walnut shells using advanced extraction techniques. Kamali et al. [16] examined the bioactive components in walnut shells using pressurized hot water extraction and pressurized liquid extraction. As a result of the study, they showed that pressurized hot water extraction exhibited the highest level of phenolic compounds and antioxidant capacity. These authors compared innovative techniques such as pressurized hot water extraction and pressurized liquid extraction instead of time-consuming traditional extraction methods.

A review of the literature on advanced extraction techniques applied to walnut shells revealed no reports on enzyme-assisted or combined extraction methods. Moreover, only a few studies focused on comparing the phenolic profiles of walnut shells as well as examining the effects of different extraction methods (PLE, UAE, EAE, and combined extraction) on the composition and content of phenolic compounds. The focus was on understanding the behavior of process parameters to identify the key characteristics of each technique, determine the optimal extraction conditions for maximizing the yield and quality of the desired compounds, and compare these results with those obtained from conventional approaches.

The aim of this research was to recover high-value functional compounds from walnut shells using innovative extraction techniques including PLE, UAE, EAE, and combined extraction (US-EAE) and to quantify the bioactive compounds using ultra-performance liquid chromatography-electrospray tandem mass spectrometry (UPLC-ESI-MS/MS).

## 2. Materials and Methods

### 2.1. Materials

Walnut (*Juglans regia* L.) plants of the Chandler variety were collected from an orchard located in Çömelek village (Mut district, Mersin province, Turkey) in October 2021, during the typical harvest period at full physiological maturity. The orchard was managed under conventional agricultural practices, including periodic irrigation and the application of inorganic fertilizers. The region is situated within the Mediterranean climate zone, which is considered favorable for walnut cultivation and is characterized by hot, dry summers and mild, wet winters.

After harvesting, the walnuts were dried at room temperature and manually shelled. The walnut shells were then ground using a centrifugal high-speed mill equipped with a 1 mm sieve (Retsch GmbH ZM 200 model, Haan, Germany). The resulting shell powder was stored in airtight glass jars under dry and dark conditions until further fractionation.

Folin–Ciocalteu phenolic reagent, gallic acid (>99%), and anhydrous sodium carbonate (%98) were supplied from Merck (Darmstadt, Germany). The 2,2-diphenyl-1-picrylhydrazyl (DPPH•), 6-hydroxy, 2,5,7,8-tetramethylchroman-2-carboxylic acid (Trolox, ≥97%), and 2,2′-azinobis-3-ethylbenzotiazoline-6-sulfonate (ABTS•) were provided from Sigma-Aldrich. NaCl, KCl, KH_2_PO_4_, and K_2_S_2_O_8_ were from Lach-Ner (Brno, Czech Republic), and Na_2_HPO_4_ was from Merck KGaA (Darmstadt, Germany). Viscozyme^®^L (a cellulolytic enzyme mixture, ≥100 FBGU/g) was sourced from Sigma-Aldrich (Bornem, Belgium).

Diatomaceous earth (100% SiO_2_, Dionex Corporation, Sunnyvale, CA, USA), cotton wool (Bella-Cotton, Toruń, Poland), ASE filters (Glass Fiber-(X)-Cellulose, Dionex Corporation, Sunnyvale, CA, USA), liquid nitrogen (AGA SIA, Riga, Latvia), carbon dioxide, and nitrogen gases (99.9%, Gaschema, Jonava region, Lithuania) were used. HPLC and analytical grade solvents were used for the extraction and analysis processes.

The polyphenols were quantified using the following analytical standards: *p*-salicylic acid, caffeic acid, and vanillin were obtained from HPC Standards (Borsdorf, Germany); acacetin, taxifolin, quercitrin, ellagic acid, and *o*-salicylic acid were obtained from TRC (Pickering, ON, Canada); and gallic acid, protocatechuic acid, isoquercitrin, naringenin, and catechin were obtained from Sigma-Aldrich Inc. (St. Louis, MO, USA). These served as external standards for UPLC-ESI–MS/MS analysis.

### 2.2. Proximate Analyses

To describe the proximate analysis of walnut shell, moisture content [17], protein content [18], crude fat content [19], ash determination [20], and crude dietary fiber content [21] were determined. Walnut shells were subjected to proximate analysis in triplicate using standard procedures.

### 2.3. Maceration (M)

Maceration in an Erlenmeyer flask (100 mL) was performed on an orbital shaker (Biosan ES-20/60) with agitation fixed on 150 rpm, at ambient temperature, 1:20 solid–solvent ratio (water and ethanol), and 1 mm particle size. The samples were collected after 24 h. After the treatment, the sample was immediately centrifuged (Orto alresa consul 22 R) at 4500 rpm for 10 min. The resulting supernatants (water-soluble fractions) were freeze-dried (−50 °C, 0.5 mbar) (MAXI lyo) or evaporated using an organic solvent in a Büchi V-850 Rotavapor R-210 (Flawil, Switzerland). The result was weighed (±0.001 g) and stored at −20 °C until further analysis.

### 2.4. Pressurized Liquid Extraction (PLE)

Pressurized liquid extraction (PLE) was conducted using ethanol (EtOH) and acetone (AC) in 66 mL extraction cells within an ASE 350 accelerated solvent extraction system manufactured by Dionex (Sunnyvale, CA, USA). In this study, water was selected as the extraction solvent for UAE, EAE, and US-EAE applications due to its sustainability, safety, and compliance with green extraction principles. However, for PLE, organic solvents such as acetone and ethanol were employed because water’s physicochemical properties (e.g., high surface tension, polarity, and heat capacity) limit its efficiency under PLE conditions, especially when processing rigid lignocellulosic matrices like walnut shells. Water may also promote hydrolysis or thermal degradation of certain phenolics at elevated PLE temperatures. Conversely, acetone and ethanol offer lower boiling points, better penetration, and more efficient mass transfer under pressurized conditions, enabling a broader extraction spectrum. This solvent selection also allowed for meaningful comparison across extraction techniques under their respective optimal operational conditions. Each cell was equipped with two cellulose filters positioned at the top and bottom, with 10 g of walnut shell inserted between them. Throughout each experiment, the following parameters were consistently maintained: pressure at 10.3 MPa, pre-heating time for 5 min, complete cell flush volume, and a nitrogen purge duration of 120 s for extract collection. The temperature during extraction was maintained at 90 °C for EtOH and 70 °C for AC, with each extraction cycle lasting 15 min, for a total of 3 cycles. Organic solvents were evaporated using a Büchi V-850 Rotavapor R-210 (Flawil, Switzerland). Following extraction, PLE-AC and PLE-EtOH extracts underwent a 10 min nitrogen flow treatment to remove solvent residues, were weighed with an accuracy of ±0.001 g, and were stored at −20 °C until further analysis.

### 2.5. Enzyme-Assisted Extraction (EAE)

Walnut shells were mixed with distilled water in a 100 mL polyethylene flat-bottom centrifugation bottle at a ratio of 1:20. Subsequently, 0.1 M HCl was added to adjust the pH to 4.5. Following this, a cellulolytic enzyme mixture, Viscozyme^®^ L, was introduced to achieve an enzyme/substrate (E/S) ratio of 0.06% *v*/*w*. The mixture was then incubated in a thermostatically controlled shaker (Bioasan ES-20/60) at 150 rpm and 45 °C for 3 h. Upon completion of the incubation period, the sample underwent centrifugation at 4500 rpm for 10 min. The resulting supernatants (water-soluble fractions) were subsequently freeze-dried at −50 °C under 0.5 mbar pressure using a MAXI lyophilizer (Telstar, Terrassa, Spain), weighed with an accuracy of ±0.001 g and stored at −20 °C until further analysis.

### 2.6. Ultrasound-Assisted Extraction (UAE)

One g of ground walnut shell powder (dry weight) was suspended in 20 mL of water. The mixture was agitated with an ultrasonic probe for 10 min. The equipment used was a Hielscher UP200Ht ultrasonic homogenizer (Hielscher Ultrasonics, Teltow, Germany) and was cooled to −15 °C with ethylene glycol in an immersion cooler (Model Julabo FT402). A 19 mm probe was immersed 1 cm deep into the sample mixture and operated. The frequency was set to 26 kHz with an output power of 200 W. After treatment, the sample was immediately centrifuged at 4500 rpm for 10 min. The resulting supernatants (water-soluble fractions) were then freeze-dried at −50 °C under 0.5 mbar pressure, weighed with an accuracy of ±0.001 g and stored at −20 °C until further analysis.

### 2.7. Combined Extraction (Ultrasound-Assisted Enzyme Extraction—US-EAE)

In this study, ultrasound-assisted enzyme extraction (US-EAE) was applied as a two-step sequential process. In the first step, walnut shell powder was subjected to a pretreatment based on the ultrasound extraction (UAE) protocol described in Section 2.6. Following the ultrasound application, enzyme-assisted extraction was performed under the optimized conditions specified in Section 2.5.

### 2.8. Determination of Extraction Yield

Extraction efficiency (%) is a parameter utilized to evaluate the efficacy of the solvent in extracting particular components from the initial material. It is quantified as the ratio of the recovered extract mass to the initial sample mass. Assessment of extraction efficiency was conducted by weighing the dry extract after drying. The yield of the extracted material was computed using the formula provided in the following Equation (1):

Yield (%) = (Weight of recovered extract)/(Weight of dry sample) × 100(1)

### 2.9. Total Phenolic Content (TPC)

The total phenolic content (TPC) of the walnut shell extracts was determined using the Folin–Ciocalteu method, following the procedure outlined by Singleton et al. [22]. Briefly, 100 µL of the sample was added to cuvettes, followed by the addition of 750 µL Folin reagent. The mixture was then incubated for 5 min before adding 600 µL of 6% sodium carbonate solution. After a 2 h incubation period, the absorbance of the mixture was measured at 760 nm using a spectrophotometer (Thermo Scientific-Genesys 50, Thermo Fisher Scientific, Waltham, MA, USA). TPC values were calculated based on the absorbance against a gallic acid calibration curve (0–80 µg/mL; R^2^ = 0.9973) and expressed as milligrams of gallic acid equivalents (GAE) per 100 g of dry weight (mg GAE/100 g dw).

### 2.10. Total Antioxidant Capacity (TAC)

#### 2.10.1. DPPH Assay

DPPH^•^ scavenging capacity was assessed following the method outlined by Brand-Williams et al. [23]. In summary, 1000 µL of DPPH methanolic solution (89.7 µmol/L, with absorption adjusted to 0.800 ± 0.010 AU at 517 nm) was combined with 500 µL of shell extract dilutions (500, 250, 100 µg/mL) or methanol (blank). After a 2 h incubation period, the absorbance was measured at 517 nm using a Thermo Scientific-Genesys 50 spectrophotometer. The scavenging activity was calculated using the following equation:

DPPH^•^ scavenging activity (%) = (A_0_ − A_s_)/A_0_) × 100 (2)

where A_0_ is the absorbance of the DPPH^•^ solution without the sample and A_s_ is the absorbance with the sample. Calibration curves were prepared using Trolox^®^ (Sigma-Aldrich, St. Louis, MO, USA) solutions at various concentrations (0–50 µmol/L; R^2^ = 0.9982). The results were expressed in milligrams of Trolox^®^ equivalents per 100 g of dry weight (mg TE/100 g dw).

#### 2.10.2. ABTS Assay

The method described by Re et al. [24] was employed in this study. Phosphate buffered saline (PBS) solution, consisting of 8.18 g NaCl, 0.27 g KH_2_PO_4_, 1.78 g Na_2_HPO_4_·2H_2_O, and 0.15 g KCl in 1 L of distilled water, was adjusted to pH 7.4 using NaOH. The ABTS^•+^ solution was prepared by combining 50 mL of ABTS (2 mM) with 200 µL of K_2_S_2_O_8_ (70 mM) and incubating the mixture in darkness for 15–16 h before use. The working solution was prepared by diluting the ABTS^•+^ solution with PBS to achieve an absorbance of 0.800 ± 0.030 at 734 nm. For the assay, 25 µL of the extract, Trolox^®^ standard solutions used for the calibration curve (150–1000 µmol/L; R^2^ = 0.9977), and a blank (methanol) were mixed with 1500 µL of the working solution. The absorbance was measured after a 2 h incubation period at 734 nm using a Thermo Scientific-Genesys 50 spectrophotometer. The radical scavenging activity was calculated using the following equation:

Scavenging activity (%) = (A_0_ − A_s_)/A_0_) × 100 (3)

where A_0_ is the absorbance of the ABTS^•+^ solution without the sample and A_s_ is the absorbance with the sample. The results were expressed in milligrams of Trolox^®^ equivalents per 100 g of dry weight (mg TE/100 g dw).

### 2.11. Polyphenol Characterization and Quantification by UPLC-ESI–MS/MS

As previously reported in the literature [25], all samples were filtered through 0.22 μm membrane filters and injected into the UPLC-ESI-MS/MS system (Shimadzu LC-MS/MS 8060, Kyoto, Japan) equipped with a C18 column (10 cm × 3 mm, 3 μm; GL Sciences, Kyoto, Japan) for quantifying polyphenols.

The injection volume was set to 10 μL, with the flow rate adjusted to 0.4 mL/min. Mobile phases A and B were MQ water and formic acid (1000:1, *v*/*v*) and acetonitrile and formic acid (1000:1, *v*/*v*), respectively. The gradient elution profile was programmed as follows: 0 min, 20% B; 0.0–0.5 min, 20% B, isocratic; 0.5–7.0 min, 20–50% B, linear; 7.0–12.0 min, 50–95% B, linear; 12.0–12.1 min, 95–20% B, linear; 12.1–15.0 min, 20% B, isocratic. The column and autosampler temperatures were maintained at 40 °C and 10 °C, respectively. The instrument parameters were as follows: the nebulizing gas (N_2_) flow rate was set to 3.0 L/min, while the drying gas (N_2_) flow rate was adjusted to 10.0 L/min. Additionally, the interface voltage was maintained at 4.0 kV, the desolvation line temperature was held steady at 250 °C, and the interface temperature was set to 300 °C. The heat block temperature was maintained at 400 °C. The MS/MS system operated in both negative and positive ion modes, utilizing multiple reaction monitoring (MRM) with the electrospray ionization (ESI) source.

### 2.12. Statistical Analysis

All extraction experiments and chemical composition analyses were conducted in triplicate, while antioxidant capacity measurements were performed in quadruplicate. Mean values and standard deviations were calculated using MS Excel 2016. Statistical analyses were conducted using SPSS version 28.0 (Chicago, IL, USA), where one-way ANOVA was employed to evaluate differences among groups. Tukey’s post hoc test was used to determine statistically significant differences between the means (*p* < 0.05). To assess relationships among variables, Pearson correlation coefficients were computed. Multivariate analyses, including principal component analysis (PCA) and hierarchical cluster analysis (HCA), were performed with Minitab version 21.0. PCA was utilized to detect underlying data structures and distinguish patterns across samples, while HCA grouped the samples based on their degree of similarity.

## 3. Results and Discussion

### 3.1. Proximate Analysis

The proximate chemical composition of the walnut shell is shown in Table 1. The mean composition of walnut shells was as follows: 8.50% moisture, 1.10% lipid content, 1.14% protein content, 1.42% ash, and 65.21% dietary fiber.

The proximate composition of the analyzed walnut shells was consistent with findings from previous studies. In the study conducted by Goklani et al. [26], the moisture content of walnut shells was reported as 7.85%, while the ash content was determined to be 1.18%. In another study by Hu et al. [27], the dietary fiber content of walnut shells was found to be 67.28 g/100 g. The moisture content, ash content, and dietary fiber values obtained in our study were within a similar range to those reported in the literature, indicating a correlation between our findings and previous studies. In another study, fat, protein, and ash contents were analyzed, and the results were comparable to ours [28]. Demirbas [29] reported ash contents of 2.8%, 1.4%, and 3.3% for walnut, hazelnut, and almond shells, respectively. Similarly, Gozaydin and Yuksel [30] reported the moisture, ash, and protein contents of hazelnut shells: moisture was found to be 8.93%, ash was found to be 1.48%, and protein was found to be 3.11%. In another study, the proximate composition of walnut shells was evaluated, revealing moisture and ash contents of 6.23% and 2.99%, respectively [31], which are consistent with the values obtained in our study. David [32] analyzed the moisture and ash contents resulting from the pyrolysis of walnut shells. As a result, moisture was determined as 7.80% and ash was 1.14%. The chemical composition of pecan walnut shells was investigated, and moisture, ash, protein, and fat ratios were reported as 13.1%, 1.80%, 1.81%, and 0.72%, respectively [33].

These studies demonstrated that the growth location, climatic conditions, and agricultural practices significantly influence the chemical composition of different shell varieties [33,34,35]. The findings highlight the necessity of considering environmental and agronomic factors when evaluating the proximate composition of walnut shells.

### 3.2. Effect of Conventional and Innovative Extraction Methods on Total Phenolic Content (TPC) and Total Antioxidant Capacity (TAC) of Walnut Shells

Recently, innovative extraction technologies were developed that are environmentally friendly due to reduced chemical use, shorter processing times, and better yields and extract quality. These technologies were also designed to overcome some of the limitations associated with conventional extraction methods [36,37]. Emerging extraction techniques are increasingly favored for their ability to enhance both the overall yield and selectivity of bioactive compounds extracted from plant materials [36,37].

**Figure 1:**
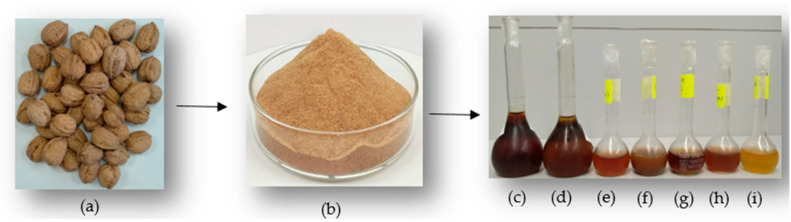
(**a**) Walnut shell, (**b**) walnut shell powder, (**c**) PLE-EtOH, (**d**) PLE-AC, (**e**) EAE, (**f**) UAE, (**g**) US-EAE, (**h**) M-Water, (**i**) M-EtOH extractions.

Both conventional and innovative extraction methods were designed and tested for the biorefinery recovery of walnut by-products, aiming to evaluate the potential for obtaining higher value-added fractions from walnut shell waste. For the phenolic component characterization of walnut shells, several extraction processes were selected, including pressurized liquid extraction (PLE), enzyme-assisted extraction (EAE), ultrasound-assisted extraction (UAE), combined ultrasound- and enzyme-assisted extraction (US-EAE), and maceration (M) techniques. Figure 1 illustrates the extracts obtained from the selected extraction methods.

#### 3.2.1. Maceration

The walnut shells were subjected to maceration using water and ethanol solutions for fractionation purposes. In this traditional extraction method, the shells were immersed in water and ethanol solutions for 24 h. TPC and TAC were analyzed as part of the control group for these extractions. The extraction yields revealed that water-soluble components accounted for 3.57 g/100 g, while ethanol-soluble components were 2.44 g/100 g. The TPC values of the extracts were measured as 529.7 mg GAE/100 g dw for the water maceration extract (M-Water) and 242.7 mg GAE/100 g dw for the ethanol maceration extract (M-EtOH) (Table 2). Antioxidant capacity measurements of the maceration extracts are also presented in Table 2. The ABTS^•+^ value for M-Water was 2275 mg TE/100 g dw, while DPPH^•^ value was 22.08 mg TE/100 g dw. Similarly, for M-EtOH, ABTS^•+^ was 901.4 mg TE/100 g dw and DPPH^•^ was 11.35 mg TE/100 g dw. The significant differences observed between the two solvents can be attributed to their distinct polarities. Water, being a highly polar solvent, is more effective in extracting hydrophilic phenolic compounds such as tannins and hydroxybenzoic acids, which are abundant in walnut shells. Ethanol, with its moderate polarity, has the ability to solubilize both hydrophilic and lipophilic compounds; however, it is less efficient than water in extracting highly polar constituents. This explains why the TPC and antioxidant capacity values of the M-Water extract were significantly higher compared to those of the M-EtOH extract. In addition to TPC and antioxidant capacity, extraction yield also varied significantly depending on the type of solvent used. Water-based maceration resulted in a higher extraction yield compared to ethanol-based extraction. This can be attributed to the ability of water to dissolve not only phenolic compounds but also other polar, non-phenolic constituents such as sugars and organic acids. However, a higher extraction yield does not necessarily indicate greater selectivity for phenolic compounds. Therefore, when evaluating extraction performance, yield values should be considered in conjunction with the qualitative composition of the extract. Similar findings were reported in the literature regarding the influence of solvent polarity on extraction yield and compound profile [38,39]. Accordingly, in conventional extraction methods such as maceration, the choice of solvent directly affects not only the quantity of extract obtained but also the chemical nature of the extracted compounds. This is one of the key factors determining the overall efficiency of the extraction method.

To further clarify the mechanisms underlying differences in extraction efficiency among conventional and advanced methods, additional factors should be considered. In maceration, the extraction process is primarily governed by passive diffusion based on concentration gradients and solvent polarity, without assistance from physical or enzymatic forces. As a result, solvent penetration into the rigid lignocellulosic matrix of walnut shells is limited, and the release of intracellular phenolics remains constrained. In contrast, PLE employs high-temperature and -pressure conditions, which reduce solvent viscosity and surface tension, improve wetting of the matrix, and enhance the desorption kinetics of solutes from plant tissues. These physicochemical changes facilitate a more efficient mass transfer and compound solubilization, particularly when sequential solvents are applied. Nonetheless, the absence of pre-disruption (e.g., by ultrasound or enzymatic action) may limit the complete accessibility of bound phenolics within the walnut shell structure. Understanding these fundamental principles is essential for interpreting the performance of extraction techniques and optimizing them for maximal bioactive recovery.

Researchers investigated the phenolic contents and antioxidant capacities of green walnuts using two maceration methods: ultrasound-assisted and conventional. They found that ultrasound-assisted maceration yielded higher levels of total phenolics, DPPH^•^, and ABTS^•+^ compared to conventional maceration. The differences in these values between the two methods were statistically significant [40].

In another study, extraction of phenolic compounds from hazelnut shells was performed using Soxhlet and maceration methods, with extraction times of 6 and 12 h, respectively [41]. While maceration avoids the high temperatures associated with Soxhlet extraction, its longer extraction duration emerged as a primary drawback. In a study by Ismail et al. [42], a comparative analysis was carried out between UAE and maceration for extracting phenolic compounds from baobab seeds. The findings indicated that UAE yielded a substantially higher TPC (418.0 mg GAE/100 g) compared to maceration (357.3 mg GAE/100 g). Thus, maceration required an extraction time of 24 h, while UAE required only 20 min. Innovative technologies were reported to both shorten extraction time and increase extraction compared to traditional methods [43].

#### 3.2.2. Pressurized Liquid Extraction (PLE)

Walnut shells were subjected to PLE using acetone and ethanol solutions for fractionation. These solvents primarily targeted high-polarity compounds with potential antioxidant properties. PLE, as an advanced alternative to traditional extraction methods, facilitates the rapid extraction of various antioxidants, including phenolic acids, flavonoids, and other polyphenols, under high-pressure and elevated-temperature conditions. This technique employs low-boiling-point solvents, such as acetone and ethanol, to enhance extraction efficiency and reduce processing time [43,44].

In this study, the residues remaining after PLE-AC extraction were subjected to further extraction using PLE-EtOH. This sequential extraction approach aimed to evaluate its effects on extract yield, TPC, and TAC. The results showed that the yield of acetone-soluble components was 1.58 g/100 g, while ethanol-soluble components yielded 2.84 g/100 g. The TPC values of the extracts were determined as 1788 mg GAE/100 g dw for PLE-EtOH and 566.8 mg GAE/100 g dw for PLE-AC (Table 2).

Antioxidant capacity measurements of the PLE extracts are also presented in Table 2. For PLE-EtOH, the ABTS^•+^ value was 6861 mg TE/100 g dw and the DPPH^•^ value was 55.16 mg TE/100 g dw. Similarly, for PLE-AC, the ABTS^•+^ value was 2201 mg TE/100 g dw while the DPPH^•^ was found to be 21.72 mg TE/100 g dw. These findings indicate that the sequential extraction strategy significantly enhanced both the extraction yield and the recovery of bioactive compounds. The higher TPC and TAC values observed in the second step (PLE-EtOH) may be attributed to the polarity-dependent solubility of phenolic compounds. Acetone, being less polar, is more effective at solubilizing moderately polar flavonoids, whereas ethanol, with its intermediate polarity and hydrogen-bonding capacity, can extract a broader range of phenolics, including more hydrophilic phenolic acids [38,39]. Additionally, the initial PLE-AC treatment may have disrupted the lignocellulosic matrix and enhanced the matrix permeability, facilitating deeper solvent penetration and improved mass transfer in the subsequent ethanol extraction step.

These synergistic effects emphasize the importance of solvent order and polarity in optimizing both the quantity and quality of phenolic-rich extracts. Therefore, extraction yield in this context not only reflects the total mass recovery but also contributes significantly to the functional potential and compositional diversity of the obtained extract.

In the literature, TPC of pistachio shells was analyzed using the PLE method. Under optimized conditions (110–150 °C, 69 bar), water extraction of pistachio shells was performed for 480 min, and a yield of 39.5 g/kg was obtained [45]. Although water was successfully applied as a green solvent in PLE for plant matrices such as pistachio shells, as demonstrated by Ersan et al. [45], water was not selected for PLE in this study due to technical and analytical considerations. Under high temperature and pressure, water may promote the hydrolysis or degradation of certain heat-sensitive phenolic compounds. Moreover, the high surface tension and strong polarity of water may limit its penetration into the dense lignocellulosic structure of walnut shells, reducing extraction efficiency. Therefore, moderately polar organic solvents such as acetone and ethanol were chosen. In particular, acetone offers a low boiling point and broad solubility range for phenolics, facilitating the efficient extraction of diverse bioactive compounds under pressurized conditions. This allowed us to assess solvent polarity effects and achieve a broader phenolic profile. Nevertheless, the potential of water-based PLE remains an important avenue for future studies aiming at greener extraction optimization. In another study, phenolic compounds and antioxidants in Brazil nut shells were analyzed using the PLE method. In this study, the highest TPC (4673 mg GAE/100 g) and antioxidant capacity (397 μmol TE/g) were achieved at 200 °C and a 50% (*v*/*v*) water/alcohol (ethanol) mixture, compared to other conditions [46]. According to these findings, the level of bioactive compounds obtained through PLE increased with rising temperatures [45,46].

Herrera et al. [14] investigated the TPC and DPPH^•^ radical scavenging activity of walnut shells using the PLE method. TPC values ranged from 5.10 to 88.82 mg GAE/g, while DPPH^•^ values ranged from 3.14 to 7.17 µg/mL, depending on the solvent ratios used during extraction. Similarly, Kamali et al. [16] analyzed the phenolic content and antioxidant capacity of walnut shells via PLE. Their results indicated a total phenolic content of 816.7 μg GAE/g dry extract and a DPPH^•^ value of 0.258 mg/mL, with the highest phenolic content and the lowest DPPH^•^ activity observed in their study.

In one of the most comprehensive studies conducted by Floegel et al. [47], the TAC of 50 antioxidant-rich fruits, vegetables, and beverages commonly consumed in the United States diet was comparatively analyzed using ABTS^•+^ and DPPH^•^ radical scavenging methods. The findings of the study revealed that the TAC of fruits and vegetables determined by the ABTS^•+^ method was significantly higher than DPPH^•^ method.

Since the ABTS^•+^ radical is soluble in both water and organic solvents, whereas the DPPH^•^ radical is soluble only in organic solvents, the DPPH^•^ method is mostly effective in measuring lipophilic components. This difference explains the higher values of ABTS^•+^ analysis, especially in the presence of hydrophilic antioxidants such as polyphenols [48,49]. Floegel et al. [47] reported that antioxidant capacity values measured by ABTS^•+^ and DPPH^•^ methods showed high correlation in fruits, but this correlation was weak in vegetables. Especially in fruits and vegetables with high pigment content (e.g., cherry, spinach, plum, red cabbage), antioxidant values measured by ABTS^•+^ were higher than those measured by DPPH^•^.

Walnut shells also structurally contain various hydrophilic antioxidants such as phenolic acids and flavonoids. This explains the more sensitive and comprehensive results of the ABTS^•+^ method in determining the antioxidant potential of walnut shell extracts. The higher antioxidant capacity values of walnut shell extracts measured by ABTS^•+^ compared to DPPH^•^ may be due to the fact that these compounds are mostly hydrophilic and to the solubility advantage of the ABTS^•+^ method. Indeed, these results are in parallel with the literature reported by Floegel et al. [47] and Ferrara et al. [50]. Hydrophilic fractions of walnut shells are known to contain structurally complex components such as sugar derivatives, phenolic compounds, and flavonoids [14]. In this study, acetone and ethanol were sequentially used for the extraction of phenolic compounds. According to the results, the PLE-EtOH extract, in particular, exhibited higher DPPH• radical scavenging activity compared to the other extraction methods. This indicates that the PLE-EtOH method has promising potential in terms of antioxidant activity. However, when evaluated in terms of overall selectivity, the PLE method demonstrated a performance comparable to that of other techniques. Therefore, PLE can be considered a promising approach for enhancing specific antioxidant responses, even though its general selectivity remains on a similar level with alternative extraction methods.

Our literature review revealed that studies on PLE methods applied to walnut shells are quite limited. Therefore, we believe this study provides a valuable contribution to the existing body of literature.

#### 3.2.3. Ultrasound-Assisted Extraction (UAE)

The results of UAE of phenolic compounds from walnut shells are presented in Table 2. The extraction conditions were standardized at 26 kHz, 200 W, and 10 min. Prolonged extraction times can result in the degradation or transformation of phenolic compounds; therefore, the optimal extraction time was determined to be 10 min. Under these conditions, the extraction yield was 4.69 g/100 g, while total phenolic content was measured as 4129 mg GAE/100 g dw. Antioxidant capacities, as assessed by ABTS^•+^ and DPPH^•^, were 11,150 and 45.25 mg/100 g dw, respectively (Table 2).

UAE enhances the solid-to-solvent diffusion rate by facilitating solvent penetration into the sample matrix. Additionally, the ultrasonic probe accelerates the mass transfer and diffusion of phenolic compounds from walnut shells into the solvent [15,51]. However, studies on UAE from walnut shells remain limited in the literature [15,51,52,53]. Han et al. [15] compared total phenolic yields from walnut shells using ultrasonic probe and ultrasonic bath extraction methods. Their results showed phenolic contents of 51.2 and 25.8 mg GAE/g dw, respectively, indicating that ultrasonic probes enhance solid–liquid contact and facilitate deeper solvent penetration into the cell matrix, thereby improving phenolic compound extraction. The antioxidant capacities of walnut shell extracts obtained by the UAE method were evaluated by DPPH^•^ and ABTS^•+^ analyses. The results revealed that the ABTS^•+^ method showed higher antioxidant capacity values compared to DPPH^•^. This suggests that the ABTS^•+^ method may be more sensitive in determining the antioxidant properties of walnut shell extracts [50].

Ziaolhagh and Zare [53] investigated total phenolic extraction from different parts of the walnut using ultrasonic pretreatment. They found that ultrasonic pretreatment improved phenolic compound extraction in all walnut parts, including walnut shells, highlighting its effectiveness as a pretreatment method. Similarly, Nour et al. [54] analyzed TPC and TAC (DPPH^•^) from walnut leaves using UAE, reporting phenolic values ranging between 2071 and 10,408 mg GAE/L and TAC (DPPH^•^) between 8.56 and 56.84 mmol Trolox^®^/L. These findings are comparable to our study, emphasizing walnut leaves as a rich source of polyphenols and highlighting the potential of UAE as a sustainable, efficient process for obtaining antioxidant compounds from plant sources.

Ganesapillai et al. [52] compared conventional extraction (Soxhlet) with innovative techniques such as ultrasound and microwave extraction for walnut shells. Their results demonstrated that innovative methods yielded higher extract quantities compared to conventional techniques. Among these, microwave extraction was identified as more efficient than ultrasound as a pretreatment method.

In light of these studies, the findings of our research align closely with the literature, further supporting the effectiveness and potential of UAE for polyphenol recovery from walnut shells.

#### 3.2.4. Enzyme-Assisted Extraction (EAE)

EAE primarily aims to employ enzymes as catalysts under optimized experimental conditions to facilitate the hydrolysis and degradation of plant cell walls, thereby enabling the release of intracellular components. The interaction between the plant cell wall and the enzyme’s active site induces a conformational change in the enzyme, which facilitates the disruption of cell wall bonds and promotes the release of bioactive components [55,56].

In this study, cellulolytic enzymes were applied to walnut shells using Viscozyme^®^ L, a multi-enzyme complex containing various carbohydrases. The EAE process was conducted with an enzyme-to-substrate (E/S) ratio of 0.06 mL/g, at 45 °C, pH 3.5, for 3 h. The total extraction yield was determined to be 7.5 g/100 g. The application of Viscozyme^®^ L significantly enhanced extraction efficiency compared to other methods. The superior extraction yield observed in the enzyme-assisted extraction (EAE) method can be attributed to the enzymatic breakdown of structural polysaccharides such as cellulose, hemicellulose, and pectin in the walnut shell matrix. The use of Viscozyme^®^ L facilitates the disruption of cell wall integrity, thereby enhancing the release of intracellular components and improving solvent accessibility. This enzymatic degradation reduces mass transfer resistance, allowing a greater proportion of soluble compounds to diffuse into the extract [55,56]. Such efficiency is particularly significant for rigid lignocellulosic materials like walnut shells, where mechanical disruption alone may not be sufficient.

TPC was 1439 mg GAE/100 g dw, while antioxidant capacities (ABTS^•+^ and DPPH^•^) were measured as 3436 and 12.30 mg/100 g dw, respectively (Table 2). Although several studies investigated EAE in various plant matrices, research focusing on walnut shells’ TPC and TAC using EAE remains limited.

Van Thanh et al. [57] investigated the total phenolic content (TPC) of extracts obtained from cashew nut testas using various extraction techniques, including enzyme-assisted extraction (EAE), ultrasound-assisted extraction (UAE), a combination of ultrasound- and enzyme-assisted extraction (U-EAE), and a combination of enzyme- and ultrasound-assisted extraction (E-UAE). Among these methods, the E-UAE technique yielded the highest TPC (263 g GAE/kg), whereas the UAE method resulted in the lowest TPC (204 g GAE/kg). These findings suggest that the enhanced extraction efficiency is primarily attributed to enzymatic cell wall degradation, which facilitates the release of phenolic compounds. Additionally, the application of ultrasonic waves is believed to increase the solvent–matrix interaction by improving the surface area contact, thereby enhancing both solubility and mass transfer of the bioactive constituents. Similar to our study, others emphasized that the combination of ultrasound- and enzyme-assisted extraction was more effective than single extraction (US or EAE) techniques in obtaining bioactive compounds from pomegranate peels [58].

Zhang et al. [59] investigated the TPC of Diaphragma juglandis fructus by enzyme and ultrasonic extraction methods. While enzyme extraction yielded 834.9 mg GAE/g, ultrasonic extraction yielded 938.9 mg GAE/g. The authors reported that ultrasound extraction recovered more total phenols than enzyme extraction. In their study, the enzyme extraction process used a methanol solution, whereas in our study, it was applied with distilled water—one of the green extraction solvents. Therefore, the selection of environmentally friendly, sustainable, and health-risk-free methods strengthens the significance of our study.

These innovative extraction methods not only reduce the necessity for hazardous solvents but also shorten the extraction duration. Additionally, as these methods are conducted under controlled-temperature conditions, they are particularly advantageous for the extraction of heat-sensitive compounds, including flavoring agents, bioactive components, pigments, and oils [56].

#### 3.2.5. Combined Ultrasound- and Enzyme-Assisted Extraction (US-EAE)

The use of innovative techniques, particularly in combination with enzymes, for the extraction of plant bioactives is a relatively novel approach that offers several advantages. Combined extraction methods not only reduce energy and solvent consumption but also enhance mass transfer, thereby facilitating faster extraction rates [60,61].

This study represents the first application of an innovative extraction method combining ultrasound and enzyme treatment (US-EAE) for the extraction of bioactive compounds from walnut shells. The results of the combined extraction are presented in Table 2. The extraction yield was determined to be 6.66 g/100 g, while the TPC was 5626 mg GAE/100 g dw. The antioxidant capacities, measured as ABTS^•+^ and DPPH^•^, were 14,479 and 45.38 mg/100 g dw, respectively (Table 2). Notably, the TPC and TAC of extracts prepared using the US-EAE technique were significantly higher (*p* < 0.05) than those obtained through single extraction methods (EAE or US).

The combined treatment of ultrasound and enzyme (US-EAE) yielded extracts with significantly higher TAC compared to those obtained through singular treatments (EAE or UAE). Among the methods evaluated, US-EAE proved to be the most efficient for extracting bioactive compounds from walnut shells, exhibiting the highest TAC. Moreover, a positive correlation was observed between the TAC of walnut shell extracts and their TPC.

Compared to UAE, the US-EAE method was significantly (*p* < 0.05) more effective, producing extracts with higher polyphenol content. Combined extraction not only enhanced bioactive compound recovery but also demonstrated superior efficiency in extracting phenolic compounds. This improvement can be attributed to the synergistic effects of ultrasonic power and enzymatic pretreatment. The ultrasonic treatment facilitated greater degradation of the walnut shell structure, while the enzymatic pretreatment increased the contact surface area between the solvent and the biomass, enabling the release of bound and free phenolics [2,62].

The utilization of UAE (7.8-fold) and US-EAE (10.6-fold) resulted in a significantly higher TPC compared to the control extract (without enzymatic and ultrasound pretreatment) (Table 2). The enzymatic pretreatment contributed to the degradation of the cellulosic network within the plant material, thereby increasing the contact surface area between the solvent and the biomass. This process also reduced the size of smaller particles and enhanced the rate of mass transfer, ultimately leading to a higher extraction yield of phenolics [2,63].

The TPC of cashew nut testa extracts was analyzed using various extraction techniques, including EAE, UAE, a combination of ultrasound- and enzyme-assisted extraction (U-EAE), and a combination of enzyme- and ultrasound-assisted extraction (E-UAE). While the TPC of single extractions yielded similar results, the combined extraction methods demonstrated significantly higher values, with U-EAE and E-UAE yielding 255 g GAE/kg and 263 g GAE/kg, respectively. These findings suggest that combined extraction methods are more effective in enhancing the recovery of phenolic compounds compared to single extractions [57]. Similarly, Patil et al. [58] reported that the combination of enzyme and ultrasound treatments exhibited superior efficiency in extracting bioactive compounds from pomegranate peels compared to individual enzyme or ultrasound treatments. This further highlights the potential of combined extraction techniques in improving the yield and efficacy of bioactive compound recovery.

The results clearly demonstrate that the mechanical spraying effect induced by ultrasonic frequency significantly enhanced the ABTS^•+^ scavenging activity of enzyme extracts. This enhancement can be attributed to the enzymatic hydrolysis of walnut shells, which facilitated the release of bound phenolic compounds. In terms of DPPH^•^ scavenging capacity, no statistically significant difference was observed between UAE and US-EAE (*p* > 0.05), while EAE yielded similar results to the control group (maceration). These findings indicate that the synergistic effect of enzymatic hydrolysis and ultrasonic pretreatment played a prominent role in enhancing ABTS^•+^ scavenging activity; however, the same synergy appeared to have limited influence on DPPH^•^ scavenging capacity. Therefore, the effectiveness of extraction methods may vary depending on the type of antioxidant assay employed.

Based on all the extraction methods evaluated, the efficiency of TPC extraction from walnut shells in this study followed the order: US-EAE > UAE > PLE-EtOH > EAE > PLE-AC > M-water > M-EtOH (*p* < 0.05). Furthermore, variations in bioactive compound content may be attributed to factors such as cultivar differences, cultivation techniques, soil characteristics, and climatic conditions [40].

### 3.3. Polyphenol Characterization and Quantification by UPLC-ESI–MS/MS

The phenolic profile of walnut shells was evaluated using seven different extraction methods. UPLC–ESI–MS/MS analysis of the walnut shell extracts identified 14 major polyphenols, including seven flavonoids and seven phenolic acids. Most polyphenols were detected in negative ionization mode, except for acacetin, which was identified in positive ionization mode. Identification was based on a detailed analysis of mass spectrometry data, fragmentation patterns, and comparisons with the existing literature (Table 3). The polyphenol content varied significantly across the extracts and was highest in pressurized liquid extraction methods. Despite the growing interest in walnut constituents, there remains a limited number of studies exploring this trend [14,27,64,65].

#### 3.3.1. Flavonoids

Seven flavonoids were detected in walnut shell samples, with taxifolin, catechin, and quercitrin identified as the major compounds, representing 17–49% of the total flavonoids (Table 4). Consistent with these findings, Liu et al. [66] also reported that taxifolin, catechin, and quercitrin were among the dominant polyphenols. In this study, quercitrin, catechin, and taxifolin were quantified in the ranges of 231.4–1221 mg/100 g, 171.8–813.5 mg/100 g, and 64.25–393.2 mg/100 g, respectively. Notably, the quercitrin content in walnut shells was higher than that reported in Diaphragma juglandis fructus [59]. As shown in Table 4, taxifolin and quercitrin were detected across all extraction methods, with higher concentrations observed in the PLE-AC method (16% and 50%, respectively; *p* < 0.05). PLE demonstrates significant advantages over conventional solvent extraction methods performed at atmospheric pressure. Pressurized solvents remain in the liquid state at temperatures substantially above their boiling points, allowing extractions at elevated temperatures. These conditions enhance the solubility of analytes and promote efficient desorption kinetics from the matrix [67]. Extraction solvents such as ethanol, acetone, and water, which are less effective at low temperatures, exhibit substantially improved efficiency at the elevated temperatures used in PLE [68,69,70]. This enhancement is attributed to the high-temperature and -pressure conditions in PLE, which improve the interaction between phenolic compounds and extraction solvents such as ethanol and acetone. Additionally, as temperature increases, the surface tension and viscosity of solvents decrease, facilitating better wetting and penetration of the matrix. This process enhances mass transfer and improves the extraction efficiency of phenolic compounds [71]. Consequently, phenolic compounds were detected in higher concentrations using PLE compared to other methods. Similar findings were reported by Pavez et al. [71], where phenolic compounds in olive pomace were more efficiently extracted using PLE than conventional methods.

Among the extraction methods, the highest catechin content in walnut shells was observed in the maceration-EtOH method (34.45%), followed by the PLE-EtOH (16.10%) and PLE-AC (13.02%) methods. A comparable trend was noted by Albuquerque et al. [72], who found a higher catechin content in *Arbutus unedo* fruit extracts using maceration compared to ultrasound and microwave extraction methods.

Furthermore, environmental factors influencing the growth and development of walnuts significantly affect the quantity and composition of walnut polyphenols. During the fruit-bearing and early development stages, the TPC and individual polyphenols peak in the green husk and shell. Therefore, young walnut fruits are preferred for examining phenolic constituents [64].

#### 3.3.2. Phenolic Acids and Other Bioactive Compounds

Phenolic acids, categorized as non-flavonoid secondary metabolites, are recognized for their antioxidative, antimutagenic, and antimicrobial properties, which contribute to the maintenance of individual health. Health effects associated with dietary phenolic acids have been demonstrated in the prevention of cardiovascular disease and cancer.

Seven phenolic acids were detected in walnut shell samples. The major compounds —ellagic acid, 4-hydroxybenzoic acid, and vanillin—were identified, representing 18–47% of total phenolic acids (Table 5). Consistent with these findings, in other reports, ellagic acid [73], vanillin [74,75], and 4-hydroxybenzoic acid [76] were also reported as dominant polyphenols. In this study, ellagic acid, 4-hydroxybenzoic acid, and vanillin were found in the ranges of 75.84–1021 mg/100 g, 163.1–596.9 mg/100 g, and 171.5–355.1 mg/100 g, respectively. Confirming the findings obtained for flavonoids, the PLE-AC method (19.85%) and the combined extraction method (21.02%) yielded higher levels of phenolic acids than the other extraction methods (*p* < 0.05), with the PLE method showing the highest concentration (1021 mg/100 g) (*p* < 0.05). In addition, phenolic acid contents differed significantly across all extractions (*p* < 0.05). Among the extraction methods, the gallic acid content in walnut shells was observed to be highest in the EAE method (1.44%), followed by the US-EAE method (1.34%) (Table 5).

The observed increase in gallic acid content can be attributed to the ability of enzymatic extraction, conducted under optimized experimental conditions, to facilitate the hydrolysis of plant cell walls. The interaction between the enzyme’s active site and the plant cell wall components induces a conformational change in the enzyme, promoting the disruption of cell wall linkages. This disruption enhances the release of intracellular constituents, particularly bound phenolic compounds such as gallic acid [55,56].

This mechanism highlights the role of enzymatic extraction in the selective recovery of specific phenolic compounds and suggests that the application of enzymes alone may be more effective than combined methods in releasing gallic acid from lignocellulosic matrices.

### 3.4. Chemometric Analysis

#### 3.4.1. Correlation Coefficient

Correlation analysis is crucial for understanding the interactions between variables and identifying indirect relationships [77]. Findings from this analysis contribute to a better understanding of the effects of walnut shells on TPC, TAC (DDPH^•^ and ABTS^•+^), and individual phenolic compounds.

**Figure 2:**
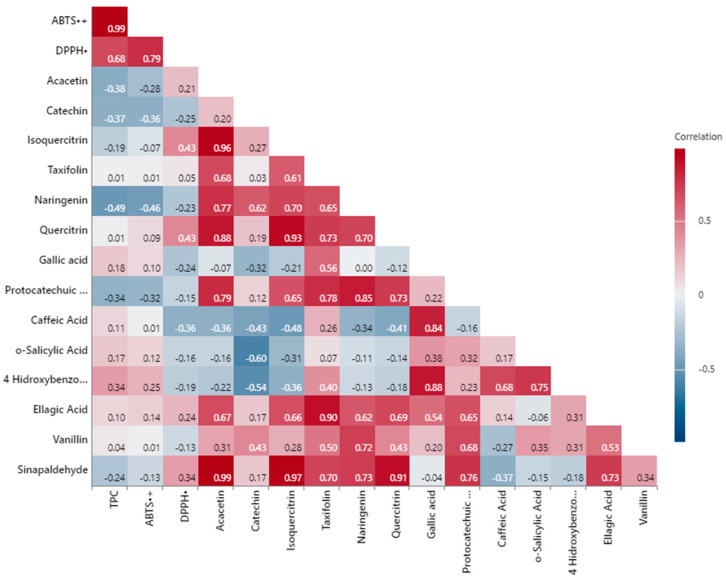
Pearson correlation analyses.

Figure 2 shows the comprehensive examination of the interrelationships among TPC, TAC, and individual phenolic compounds of walnut shell extracts. Several parameters exhibited statistically significant strong positive and negative correlations.

According to the analysis, the strongest positive relationship among the antioxidant capacity parameters was observed between TPC and ABTS^•+^, with an R^2^ value of 0.99 (*p* < 0.01). Similarly, ABTS^•+^ and DPPH^•^ exhibited a strong positive correlation (R^2^ = 0.79, *p* < 0.05). The correlation between TPC and DPPH^•^ was moderate and positive (R^2^ = 0.68). Comparable linear correlations were also observed in studies by Sheng et al. [64] and Shi et al. [78]. In samples with high TPC, antioxidant capacities are also high. Thus, TPC are known to be largely responsible for the antioxidant capacity of walnut shells [78].

In terms of individual phenolic compounds, a very strong positive correlation was found between acacetin and isoquercitrin (R^2^ = 0.96, *p* < 0.01). Additionally, a high positive correlation was observed between taxifolin and ellagic acid (R^2^ = 0.90, *p* < 0.01). Protocatechuic acid showed a significant positive correlation with both naringenin (R^2^ = 0.85, *p* < 0.05) and quercitrin (R^2^ = 0.73). Notably, sinapaldehyde exhibited strong correlation coefficients with several phenolics, including acacetin (R^2^ = 0.99, *p* < 0.01), isoquercitrin (R^2^ = 0.97, *p* < 0.01), quercitrin (R^2^ = 0.91, *p* < 0.01), and ellagic acid (R^2^ = 0.73), indicating co-variation among these compounds. These findings provide valuable insights into the contributions of phenolic compounds to antioxidant capacity and their interrelationships.

The difference in the correlation coefficient among the various antioxidant assays indicates that the overall antioxidant capacity cannot be assessed using a single antioxidant assay. Therefore, it is highly recommended to perform several assays using various mechanisms to fully understand the results [79]. Furthermore, it has been generally acknowledged in earlier research that the Folin–Ciocalteu technique is not exclusive to TPC analysis. Reducing substances can influence the analysis and cause an overestimation of the phenolic concentration. Examples of these agents are citric acid, ascorbic acid, simple sugars, and certain amino acids [49]. These results are also in agreement with other published studies, in which a positive correlation between TPC and TAC was observed [80].

Considering the extensive variety of flavonoids (over 4000 identified types) [81], additional research is necessary to gain deeper insights into the structure–activity relationships specific to walnut shells. In addition, the growth and development characteristics of walnuts vary in different environments, affecting the content and composition of walnut polyphenols.

#### 3.4.2. Principal Component Analysis (PCA)

PCA serves as a technique to simplify complex datasets by decreasing the number of variables and to facilitate a more meaningful interpretation of the results. The number of factors to be retained in a statistical analysis depends not only on the size of the dataset but also on the total amount of variance explained [82].

**Figure 3:**
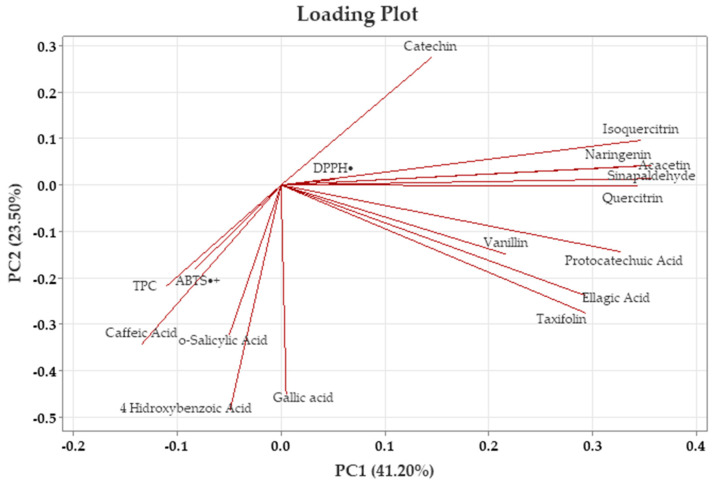
Principal component analysis (PCA) loading plot of walnut shell extracts.

Figure 2 presents the loading plot, which shows the contributions of variables such as TPC, TAC, and individual phenolic compounds to the principal component scores. In contrast, Figure 3 displays the score plot, reflecting the distribution of the analyzed samples, with each point representing an individual sample. To ensure analytical reliability in PCA, only components with eigenvalues greater than 1.00 were considered.

According to the data given in Table 6, the positive and negative contributions of the variables to PC1 and PC2 components help identify the major sources of variation in terms of TPC, TAC, and individual phenolic compounds. In PCA, only the components with eigenvalues greater than 1.00 were selected to maintain the reliability of the analysis. The results showed the first five principal components, representing 41.20%, 23.50%, 18.00%, 8.50%, and 7.40% of the variance, respectively, resulting in a total explained variance of 98.70% (Table 6). The first principal component (PC1), accounting for 41.20% of the total variance, was predominantly characterized by high positive loadings of individual phenolic compounds such as acacetin (0.945), sinapaldehyde (0.943), isoquercitrin (0.916), quercitrin (0.907), naringenin (0.906), protocatechuic acid (0.865), taxifolin (0.776), and ellagic acid (0.774). Vanillin also contributed positively to PC1 with a moderate loading (0.574). In contrast, catechin exhibited a weak positive loading (0.382), while caffeic acid showed a negative contribution to PC1 (−0.356).

The second principal component (PC2), which explains 23.50% of the total variance, showed a high positive relationship with 4-hydroxybenzoic acid (0.970). Similarly, gallic acid (0.899) exhibited high positive loadings. Caffeic acid (0.686), *o*-salicylic acid (0.642), and taxifolin (0.550) made moderate positive contributions, while catechin (−0.547) made a moderate negative contribution. In addition, ellagic acid (0.475), TPC (0.433) and ABTS^•+^ (0.361) showed a weak positive relationship.

The first two components cumulatively explained 64.70% of the total variance, being decisive in the variation in both phenolic compounds and antioxidant properties, adequately representing the main sources of variation in the analyzed traits. These components successfully represented the main variation observed in the evaluated properties. Additionally, an increase in vector direction reflected higher values of the associated traits, helping to distinguish the samples, while movement in the reverse direction indicated a negative association among these characteristics.

**Figure 4:**
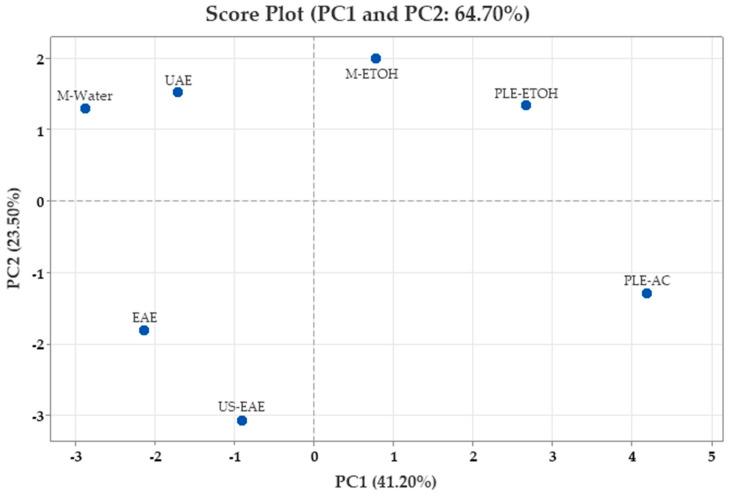
Principal component analysis (PCA) score plot of walnut shell extracts.

The PCA score plot presented in Figure 4 shows the position of walnut shells along the PC1 and PC2 axes for different extraction methods. The loading plots (Figure 3) provide important information on the main components underlying the chemical variation among the different extraction techniques. According to the loading plot, isoquercitrin, catechin, acacetin, sinapaldehyde, naringenin, quercitrin, taxifolin, protocatechuic acid, ellagic acid, and vanillin were among the variables with the highest positive contribution to the PC1 axis; these variables are grouped on the right axis in the PCA plane. On the other hand, TPC, ABTS^•+^, caffeic acid, 4-Hydroxybenzoic acid, and *o*-salicylic acid contributed negatively to PC1. DPPH^•^ and gallic acid are located near the center of the PCA plane, and their contributions to the PC1 axis were weak. While TPC, ABTS^•+^, gallic acid, caffeic acid, *o*-salicylic acid, and 4-Hydroxybenzoic acid were among the variables that contributed positively to the PC2 axis, catechin, DPPH^•^, acacetin, isoquercitrin, naringenin, and sinapaldehyde had a negative effect on this axis. This indicates that flavonoid compounds played an important role in shaping the extract properties.

When the score graph was evaluated, it was observed that PLE-AC, PLE-ETOH, and M-ETOH samples contributed positively to the PC1 component. This revealed that these extraction methods were more efficient in obtaining compounds rich in flavonoid derivatives. On the other hand, M-Water, UAE, EAE, and US-EAE samples were found to be negatively positioned on the PC1 axis. The extracts in this group were especially rich in phenolic acids, TPC, and ABTS^•+^ values.

This distribution is directly related to the predominance of the parameters that load positively or negatively on PC1 in the phenolic component profiles of the samples. In other words, the PC1 axis provides the separation of extracts according to flavonoid or phenolic acid content. On the PC2 axis, M-EtOH, PLE-EtOH, M-Water, and UAE samples are positively orientated, while PLE-AC, EAE, and US-EAE samples are negatively orientated. In particular, the PLE-AC method revealed an extract profile containing a high polyphenol load but a high proportion of components with low levels of DPPH^•^.

These findings indicate that the extracts were significantly grouped according to their phenolic compounds and antioxidant capacity levels. The combined evaluation of PCA loading and score plots revealed that the extraction methods were clearly differentiated according to phenolic compound profiles and especially that the flavonoid and phenolic acid groups were represented on different axes. In conclusion, these analyses show that PCA is a powerful and descriptive statistical tool for comparing extraction methods and classifying the obtained products according to their functional properties.

#### 3.4.3. Hierarchical Cluster Analysis (HCA)

**Figure 5:**
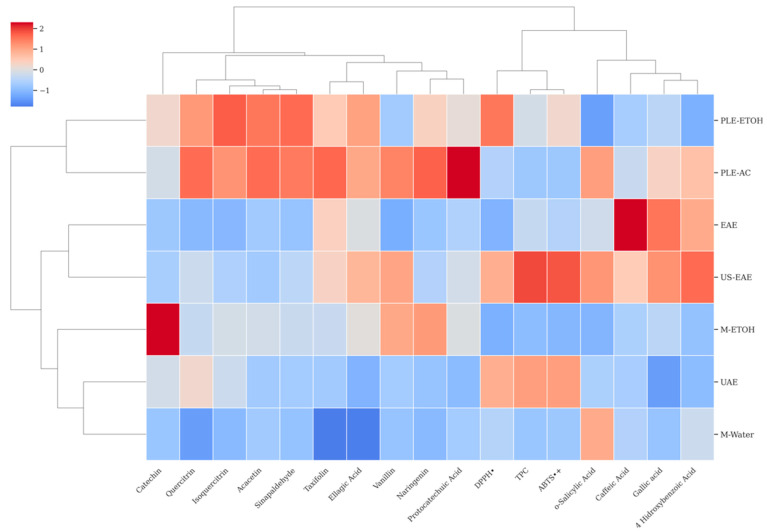
Cluster map of walnut shell extracts.

HCA is a structured method used to detect inherent groupings among samples by analyzing their measured characteristics. This approach begins with standardizing the data, followed by generating a dendrogram that illustrates the relationships, highlighting both similarities and differences among the samples [83]. As shown in Figure 5, the HCA results obtained for the dataset variables are visualized through a clustermap representation. This analysis groups the samples (rows) and the variables (columns) at the same time, allowing for a more thorough assessment of the dataset’s multivariate structure. Unlike approaches that rely solely on correlation matrices, HCA provides both statistical and visual insights into sample-level similarities and variable-based structural relationships. In this study, HCA was effectively employed to cluster the TPC, TAC (DPPH^•^ and ABTS^•+^), and individual phenolic compounds derived from walnut shells subjected to various extraction techniques. The dendrogram presented in Figure 5 offers a comparative assessment of how different extraction methods influence the chemical profiles of walnut shell extracts.

As a result of the HCA, the walnut shell extracts obtained through different extraction techniques were grouped into three main clusters. The first cluster comprised M-Water, UAE, and M-ETOH; the second cluster included US-EAE and EAE; and the third cluster consisted of PLE-AC and PLE-ETOH extractions.

These findings indicate that the applied extraction techniques have a decisive impact on both the diversity and quantity of bioactive compounds recovered from walnut shells. The clustering of conventional methods (M-Water and M-EtOH) within the same group suggests a high degree of similarity in the chemical composition of the extracts obtained by these techniques. In contrast, the grouping of innovative extraction techniques (US-EAE, EAE, PLE-AC, and PLE-ETOH) into separate clusters demonstrates their distinct influence on the extraction of individual phenolic compounds and their potential to yield more characteristic chemical profiles.

The consistency between this clustering pattern and the results of the PCA further reinforces the reliability and robustness of the multivariate statistical findings. Therefore, in the selection of extraction techniques, both the targeted functional compound profile and the outcomes of statistical modeling should be jointly considered.

The clustering analysis of all variables revealed three distinct groups. The first main cluster comprised flavonoid compounds, including catechin, quercitrin, isoquercitrin, acacetin, sinapaldehyde, taxifolin, and ellagic acid, as well as vanillin, naringenin, and protocatechuic acid. The second cluster consisted of antioxidant parameters TPC, DPPH^•^, and ABTS^•+^, while the third cluster was composed of phenolic acids, such as *o*-salicylic acid, caffeic acid, gallic acid, and 4-hydroxybenzoic acid.

A more detailed examination of the clustermap revealed that TPC, DPPH^•^, ABTS^•+^, *o*-salicylic acid, caffeic acid, gallic acid, and 4-hydroxybenzoic acid were closely located, based on Ward’s linkage method and Euclidean distance. This close grouping highlights the strong discriminative power of TPC, TAC, and specific phenolic acids in characterizing the chemical profiles of walnut shell extracts. Additionally, the co-localization of these variables within the same subclusters suggests a functional relationship between phenolic compounds and biological activities, supporting the findings obtained from PCA.

In conclusion, both HCA and PCA analyses clearly demonstrated the significant influence of extraction techniques on the phenolic composition of walnut shell extracts and indicated that multivariate statistical approaches can be effectively used in a complementary manner for the comprehensive characterization of extract profiles.

## 4. Conclusions

This study comprehensively evaluated the effects of different extraction techniques on the recovery of phenolic compounds and antioxidant properties from walnut shells. The results demonstrated that both the TPC and TAC were significantly influenced by the applied extraction methods. Among the evaluated techniques, US-EAE and PLE yielded the highest levels of phenolic content and antioxidant activity, highlighting their potential as effective and sustainable methods for the valorization of walnut shell by-products.

The phenolic composition varied considerably depending on the extraction technique, with taxifolin, catechin, and quercitrin emerging as the predominant flavonoids. Advanced statistical approaches such as PCA and HCA effectively discriminated the extracts based on their bioactive profiles, underlining the potential of multivariate analyses in understanding the relationships among extraction methods, chemical composition, and antioxidant potential.

Although the findings are promising, it is important to recognize that the extraction processes were carried out under laboratory-scale conditions. To ensure the practical applicability of these methods, especially for industrial biorefinery applications, further validation using pilot- and industrial-scale equipment is essential.

Scaling up the extraction processes may introduce new challenges related to energy consumption, solvent recovery, process efficiency, and economic feasibility. Therefore, future studies should focus on the techno-economic evaluation and process optimization at industrial scale, alongside assessing the stability and functionality of the recovered extracts in real food or nutraceutical formulations.

In summary, this study contributes to the growing body of knowledge on green extraction methods and their role in valorizing agri-food wastes. It provides a scientific basis for the development of functional ingredients from walnut shells and encourages the implementation of sustainable technologies in food and nutraceutical industries.

## Figures and Tables

**Table 1:** Proximate analysis of walnut shell.

Sample	Moisture Content (%)	Lipid Content (%)	Protein Content (%)	Ash Content (%)	Dietary Fiber (g/100 g)
Walnut Shell	8.50 ± 0.14	1.10 ± 0.21	1.14 ± 0.04	1.42 ± 0.02	65.21 ± 0.27

**Table 2:** Total phenolic content (TPC) and total antioxidant capacity (TAC) of extracts.

Assay	*Extracts*						
	PLE-EtOH	PLE-AC	EAE	UAE	US-EAE	M-EtOH	M-Water
**Yield g/100 g**	2.84 ± 0.03 ^e^	1.58 ± 0.04 ^f^	7.50 ± 0.22 ^a^	4.69 ± 0.14 ^c^	6.66 ± 0.16 ^b^	2.44 ± 0.02 ^e^	3.57 ± 0.02 ^d^
**TPC** **mg GAE/100 g dw**	1788 ± 54 ^c^	566.8 ± 20.0 ^e^	1439 ±24 ^d^	4129 ±93 ^b^	5625 ±76 ^a^	242.7 ± 7.1 ^f^	529.7 ± 14.6 ^e^
**ABTS** **mg TE/100 g dw**	6861 ± 101 ^c^	2201 ± 57 ^e^	3436 ± 85 ^d^	11,150 ± 61 ^b^	14,478 ± 89 ^a^	901.4 ± 35.1 ^f^	2275 ± 83 ^e^
**DPPH** **mg TE/100 g dw**	55.16 ± 0.45 ^a^	21.72 ± 0.26 ^c^	12.30 ± 0.32 ^d^	45.25 ± 0.82 ^b^	45.38 ± 0.75 ^b^	11.35 ± 0.23 ^d^	22.08 ± 0.42 ^c^

Different letters in the rows represent statistically significant differences (*p* < 0.05).

**Table 3:** Method parameters used for the identification of polyphenols.

Compounds	Retention Time (RT)	Ionization Mode	Mass (*m*/*z*)	Main Fragment (*m*/*z*)	Other Fragmental Ions (*m*/*z*)
***Flavonoids***					
Catechin	2.16	ESI−	289.1	205.1	145.1
Isoquercitrin	3.45	ESI−	463.1	300.0	271.0
Taxifolin	3.87	ESI−	303.1	285.1	125.0
Quercitrin	4.05	ESI−	447.1	300.0	271.0
Sinapaldehyde	4.45	ESI−	206.9	192.1	177.1
Naringenin	6.83	ESI−	270.9	151.0	119.1
Acacetin	9.46	ESI+	285.0	242.0	153.0
***Phenolic acids and other bioactive compounds***					
Gallic acid	1.74	ESI−	168.9	125.1	169.00; 79.00
Protocatechuic Acid	1.99	ESI−	152.9	109.0	90.9
4-Hydroxybenzoic Acid	2.60	ESI−	137.0	93.0	65.1; 75.1
Caffeic Acid	2.67	ESI−	178.8	135.1	89.2
Ellagic Acid	3.40	ESI−	301.0	284.0	300.0
Vanillin	3.55	ESI−	151.0	136.1	92.1
*o*-Salicylic Acid	5.06	ESI−	137.1	93.0	65.0; 75.0

**Table 4:** Changes in the flavonoid content of walnut shells obtained under different extraction conditions.

Compounds (mg/100 g)	US-EAE	UAE	EAE	PLE-ETOH	PLE-AC	M-Water	M-ETOH
Acacetin	ND	ND	ND	205.5 ± 5.7 ^a^	214.2 ± 3.9 ^a^	ND	51.77 ± 0.44 ^b^
Catechin	206.0 ± 3.9 ^c^	306.6 ± 1.4 ^b^	175.6 ± 1.4 ^c^	380.2 ± 1.1 ^b^	307.5 ± 2.3 ^b^	171.8 ± 1.3 ^c^	813.5 ± 5.8 ^a^
Isoquercitrin	13.83 ±0.45 ^d^	18.44 ± 0.51 ^c^	7.65 ± 0.45 ^e^	45.95 ± 1.57 ^a^	38.75 ± 1.91 ^b^	8.25 ± 3.57 ^e^	20.22 ± 1.92 ^c^
Taxifolin	266.3 ± 8.9 ^b^	165.4 ± 1.8 ^d^	271.6 ± 5.1 ^b^	282.6 ± 10.2 ^b^	393.2 ± 2.9 ^a^	64.25 ± 3.79 ^e^	207.5 ± 1.7 ^c^
Quercitrin	605.7 ± 18.6 ^d^	754.7 ± 29.6 ^c^	352.5 ± 12.4 ^e^	1073 ± 46 ^b^	1221 ± 52 ^a^	231.4 ± 6.9 ^f^	585.6 ± 15.5 ^d^
Sinapaldehyde	74.98 ± 0.54 ^b^	51.69 ± 1.59 ^c^	36.22 ± 1.27 ^cd^	260.8 ± 10.1 ^a^	247.3 ± 12.4 ^a^	34.31 ± 1.66 ^d^	87.32 ± 3.4 ^b^
Naringenin	2.99 ± 0.08 ^d^	1.15 ± 0.04 ^ef^	1.54 ± 0.02 ^e^	8.29 ± 0.88 ^c^	16.32 ± 0.38 ^a^	0.42 ± 0.01 ^f^	12.95 ± 0.40 ^b^

Different letters in the rows represent statistically significant differences (*p* < 0.05).

**Table 5:** Changes in the phenolic acids and other bioactive compound contents in walnut shells obtained under different extraction conditions.

Compounds (mg/100 g)	US-EAE	UAE	EAE	PLE-ETOH	PLE-AC	M-Water	M-ETOH
Gallic acid	131.7 ± 3.5 ^b^	39.04 ± 1.74 ^f^	141.7 ± 4.5 ^a^	72.36 ± 3.03 ^d^	99.44 ± 1.57 ^c^	58.36 ± 0.70 ^e^	72.08 ± 0.66 ^d^
Protocatechuic Acid	14.86 ± 1.34 ^bcd^	10.87 ± 0.43 ^e^	12.87 ± 0.50 ^cde^	16.14 ± 0.88 ^b^	26.83 ± 0.02 ^a^	12.31 ± 0.16 ^de^	15.53 ± 0.33 ^bc^
Caffeic Acid	56.38 ± 0.66 ^b^	8.95 ± 0.05 ^e^	134.41 ± 2.83 ^a^	8.40 ± 0.10 ^e^	24.07 ± 0.29 ^c^	13.72 ± 0.32 ^d^	10.82 ± 0.08 ^de^
*o*-Salicylic Acid	7.01 ± 0.24 ^a^	3.92 ± 0.36 ^bc^	4.61 ± 0.04 ^b^	2.61 ± 0.04 ^d^	6.86 ± 0.11 ^a^	6.63 ± 0.35 ^a^	3.10 ± 0.12 ^cd^
4-Hydroxybenzoic Acid	596.9 ± 4.5 ^a^	188.8 ± 2.1 ^e^	492.7 ± 4.1 ^b^	163.1 ± 4.3 ^f^	439.6 ± 5.7 ^c^	300.5 ± 6.7 ^d^	200.3 ± 1.3 ^e^
Ellagic Acid	929.5 ± 10.1 ^b^	287.3 ± 5.9 ^d^	654.2 ± 2.62 ^c^	1021 ± 36 ^a^	999.2 ± 20.3	75.84 ± 0.92 ^e^	668.4 ± 4.5 ^c^
Vanillin	330.1 ± 13.3 ^ab^	207.2 ± 1.4 ^c^	171.5 ± 6.9 ^d^	205.8 ± 4.8 ^c^	355.1 ± 2.1 ^a^	196.2 ± 6.7 ^cd^	328.3 ± 1.3 ^b^

Different letters in the rows represent statistically significant differences (*p* < 0.05).

**Table 6:** Variance ratios explained by the first five principal components.

Variables	PC1	PC2	PC3	PC4	PC5
Eigenvalue	7.002	4.002	3.063	1.446	1.259
Percentage %	41.20	23.50	18.00	8.50	7.40
Cumulative	41.20	64.70	82.80	91.30	98.70
TPC	−0.291	0.433	0.785	0.197	0.251
ABTS**^•+^**	−0.219	0.361	0.869	0.163	0.196
DPPH**^•^**	0.136	−0.024	0.978	−0.045	−0.061
Acacetin	0.945	−0.085	0.047	−0.183	−0.243
Catechin	0.382	−0.547	−0.276	0.184	0.665
Isoquercitrin	0.916	−0.193	0.286	−0.164	−0.114
Taxifolin	0.776	0.550	−0.021	−0.207	0.122
Naringenin	0.906	−0.081	−0.338	0.183	0.155
Quercitrin	0.907	0.003	0.342	−0.006	−0.077
Gallic acid	0.013	0.899	−0.248	−0.258	0.203
Protocatechuic Acid	0.865	0.287	−0.263	0.194	−0.230
Caffeic Acid	−0.356	0.686	−0.305	−0.524	0.149
*o*-Salicylic Acid	−0.131	0.642	−0.144	0.535	−0.510
4-Hydroxybenzoic Acid	−0.130	0.970	−0.155	0.119	−0.043
Ellagic Acid	0.774	0.475	0.133	−0.194	0.297
Vanillin	0.574	0.298	−0.159	0.682	0.301
Sinapaldehyde	0.943	−0.027	0.179	−0.163	−0.197

## Data Availability

The original contributions presented in this study are included in the article. Further inquiries can be directed to the corresponding author.
